# Genetic Mutations of Pancreatic Cancer and Genetically Engineered Mouse Models

**DOI:** 10.3390/cancers14010071

**Published:** 2021-12-24

**Authors:** Yuriko Saiki, Can Jiang, Masaki Ohmuraya, Toru Furukawa

**Affiliations:** 1 Department of Investigative Pathology, Tohoku University Graduate School of Medicine, Sendai 980-8575, Japan; ysaiki@med.tohoku.ac.jp (Y.S.); can.jiang.t3@dc.tohoku.ac.jp (C.J.); 2 Department of Genetics, Hyogo College of Medicine, Hyogo 663-8501, Japan; ohmuraya@hyo-med.ac.jp

**Keywords:** KRAS, GEMM, PDAC

## Abstract

**Simple Summary:**

Pancreatic ductal adenocarcinoma (PDAC) is a highly aggressive malignancy. Recent multi-gene analysis approaches such as next-generation sequencing have provided useful information on the molecular characterization of pancreatic tumors. Different types of pancreatic cancer and precursor lesions are characterized by specific molecular alterations. Genetically engineered mouse models (GEMMs) of PDAC are useful tools to understand the roles of altered genes. Most GEMMs are driven by oncogenic Kras, and can recapitulate the histological and molecular hallmarks of human PDAC and comparable precursor lesions. In this review, we summarize the main molecular alterations found in pancreatic neoplasms and GEMMs developed based on these alterations.

**Abstract:**

Pancreatic ductal adenocarcinoma (PDAC) is a highly aggressive malignancy, and the seventh leading cause of cancer-related deaths worldwide. An improved understanding of tumor biology and novel therapeutic discoveries are needed to improve overall survival. Recent multi-gene analysis approaches such as next-generation sequencing have provided useful information on the molecular characterization of pancreatic tumors. Different types of pancreatic cancer and precursor lesions are characterized by specific molecular alterations. Genetically engineered mouse models (GEMMs) of PDAC are useful to understand the roles of altered genes. Most GEMMs are driven by oncogenic Kras, and can recapitulate the histological and molecular hallmarks of human PDAC and comparable precursor lesions. Advanced GEMMs permit the temporally and spatially controlled manipulation of multiple target genes using a dual-recombinase system or CRISPR/Cas9 gene editing. GEMMs that express fluorescent proteins allow cell lineage tracing to follow tumor growth and metastasis to understand the contribution of different cell types in cancer progression. GEMMs are widely used for therapeutic optimization. In this review, we summarize the main molecular alterations found in pancreatic neoplasms, developed GEMMs, and the contribution of GEMMs to the current understanding of PDAC pathobiology. Furthermore, we attempted to modify the categorization of altered driver genes according to the most updated findings.

## 1. Introduction

Pancreatic cancer is one of the most lethal cancers, with a rising incidence, particularly in developed countries. Poor prognosis may be due to a lack of investigative approaches and the deadly characteristics of the cancer. Pancreatic cancer is the fourth leading cause of cancer-related deaths in Japan [1] and seventh worldwide [2]. It is projected to become the third leading cause of cancer-related deaths by 2025 in Europe and the second leading cause by 2030 in the United States [3,4].

Pancreatic ductal adenocarcinoma (PDAC) is the most common form of pancreatic cancer. PDAC has both genetic and epigenetic aspects of its formation and progression. The PDAC genome, which is the focus of this review, has been well studied. Several sequencing studies have confirmed the four main drivers (*KRAS*, *CDKN2A*, *TP53* and *SMAD4*) of PDAC identified prior to the era of next-generation sequencing [5]. Whole-exome sequencing identified additional signaling pathways. Jones et al. identified twelve genetically altered core signaling pathways, including apoptosis, DNA damage control, regulation of G1/S phase transition, hedgehog signaling, homophilic cell adhesion, integrin signaling, c-Jun N-terminal kinase signaling, KRAS signaling, regulation of invasion, small GTPase-dependent signaling (other than KRAS), transforming growth factor-beta (TGF-β) signaling, and Wnt/Notch signaling [6]. The authors suggested that dysregulation of these core pathways could explain the major features of pancreatic tumorigenesis. Hayashi et al. classified commonly altered driver genes into nine categories, including RNA splicing, mitogen-activated protein kinase/extracellular signal-regulated kinase (MAPK-ERK) signaling, TGFβ signaling, C1/S checkpoint, genome stability, stem cell renewal, DNA damage repair, switch/sucrose non-fermentable (SW1/SNF) complex, and complexes of proteins associated with Set1 (COMPASS) [7].

PDAC is genetically homogeneous with recurrent mutations in four genes. However, it is clinically heterogeneous. Chan-Seng-Yue et al. analyzed a dataset of whole genomes and transcriptomes generated from purified tumor cells using laser capture microdissection [8]. The cohort was segregated into five molecular subtypes of PDAC, namely basal-like-A, basal-like-B, hybrid, classical-A, and classical-B. The authors demonstrated that molecular subtypes are linked to specific copy number aberrations in genes such as mutant *KRAS* and *GATA6*, and disease heterogeneity is due to ongoing genomic instability during progression [8].

Five precursor lesions of PDAC have been recognized: pancreatic intraepithelial neoplasia (PanIN) [9], intraductal papillary mucinous neoplasm (IPMN) [10], intraductal oncocytic papillary neoplasm (IOPN) [11], pancreatic intraductal tubulopapillary neoplasm (ITPN) [12], and mucinous cystic neoplasm (MCN) [13]. PDACs predominantly originate from PanIN, and are commonly recognized as a conventional type of PDAC. However, some of PDACs can also be derived from other precursor lesions. IOPN is classically considered as one of the subtypes of IPMN. However, it is now recognized as a distinct entity [14]. The most frequent mutations of PDAC are *KRAS*, *CDKN2A*, *SMAD4*, and *TP53* [6]. The precursor lesions of PDAC also harbor these mutations [15]. Although IOPNs and ITPNs typically lack these mutations, limited data are available concerning pathogenesis [16,17].

Genetically engineered mouse models (GEMMs) of PDAC have improved our understanding of this deadly disease. Since an oncogenic *KRAS* mutation is the most frequent event of pancreatic cancer, most GEMMs are based on *Kras* mutation (Table 1).

**Figure 1:**
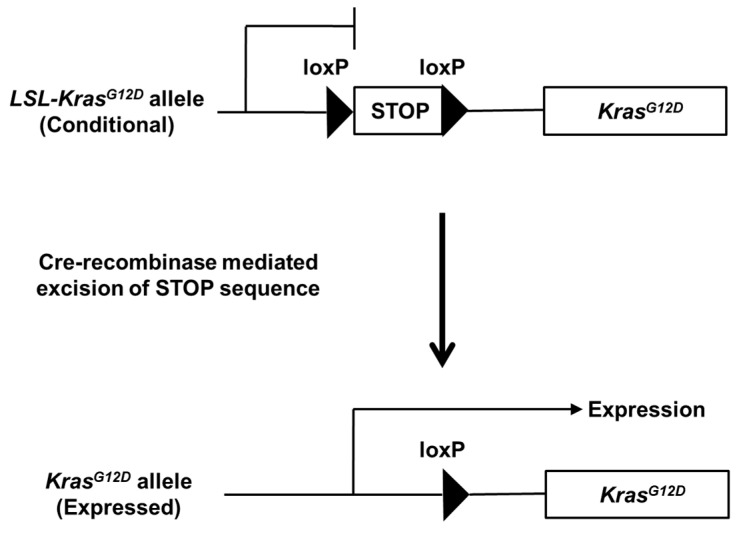
Targeting endogenous Kras^G12D^ expression to the mouse pancreas. Conditional *LSL-Kras^G12D^* allele and generation of expressed *Kras^G12D^* allele after Cre recombinase-mediated excision of STOP sequence.

The study of the Cre-activated *Kras^G12D^* allele (*LSL-Kras^G12D^*) was first constructed by Jackson et al. [43]. The endogenous *Kras* locus was targeted, and a genetic inhibitory element flanked by the loxP site was inserted upstream of the oncogenic *Kras* gene, containing a mutation of codon 12 (G12D). To facilitate a better understanding of endogenous Kras^G12D^ expression, a schematic regulation flow is depicted in Figure 1. By crossing these mice with mice expressing Cre recombinase under a specific promoter, the stop element was removed, and Kras^G12D^ was expressed in the restricted cell lineage. The transcription factors Pdx1, Ptf1a and Sox9 are expressed in early pancreatic progenitor cells. Their pancreas-specific promoters are used to conditionally express Kras^G12D^ in the pancreatic cell lineage. Koop et.al. generated *Sox9-CreER^T2^* mice by expressing a tamoxifen-inducible form of Cre recombinase in the adult pancreatic duct in the mice [44]. Using these mice, tamoxifen administration resulted in the expression of oncogenic Kras^G12D^ in adult pancreatic ducts [18].

## 2. Altered Pathways of Pancreatic Cancer and GEMM

### 2.1. KRAS Pathway

**Figure 2:**
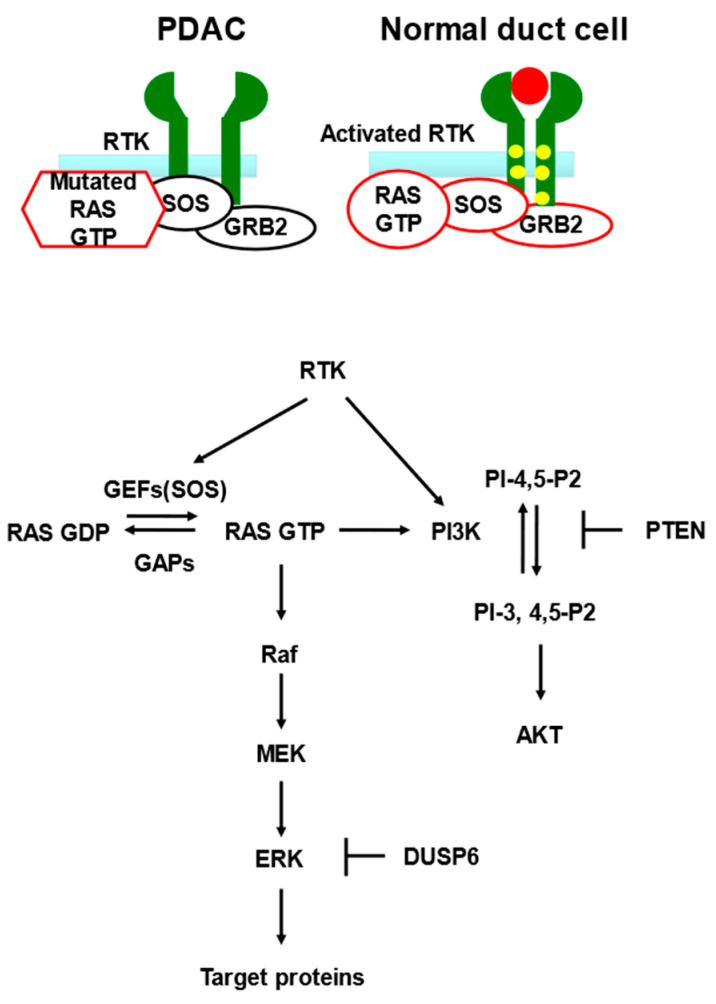
The RAF/MEK/ERK and PI3K/AKT signaling pathways. Receptor tyrosine kinases (RTKs) integrate signals from extracellular growth factors to recruit guanine nucleotide exchange factors (GEFs), which promote the exchange of GDP for GTP on Ras. In its GTP-bound state, RAS activates downstream effector pathways, including the RAF/MEK/ERK and PI3K/AKT pathways. GTPase activating proteins (GAPs) promote the hydrolysis of Ras-GTP to Ras-GDP, thereby downregulating both Raf/MAPK and PI3K signaling. The PI3K pathway is negatively regulated by phosphatases such as PTEN. Dual-specificity phosphatase 6 (DUSP6) negatively regulates MAPK signaling by dephosphorylating ERK. Mutant forms of RAS are resistant to GAP-mediated GTPase stimulation and are locked permanently in the GTP-bound active state, resulting in continuous stimulation of the MAPK cascade without ligand binding. A solid red circle indicates a ligand for RTK.

KRAS is a membrane-bound guanosine triphosphate (GTP) binding protein and that mediates various cellular functions such as cell survival, proliferation, and cell motility. The intracellular signaling pathway is caused by changes in the active and inactive states of KRAS. KRASs is activated when bound to GTP by nucleotide exchange factors (GEFs), and inactivated when bound to GDP by GTPase-activating proteins (GAPs). In its active state, KRAS activates the RAF/MEK/ERK and phosphoinositide 3-kinase/protein kinase B (PI3K/AKT) signaling pathways (Figure 2) [45].

The mutational activation of RAS in human cancer was first demonstrated in 1982 [46]. Oncogenic alteration of the *KRAS* gene occurs in approximately 30% of human cancers [6]. A cohort study of 109 pancreatic ductal carcinomas subjected to whole-exome sequencing detected mutations in the *KRAS* gene in 92% of the cases. Most *KRAS* mutations occurred in codon 12 [47]. Oncogenic mutant KRAS is resistant to downregulation by GAP-mediated hydrolysis and binds to effector proteins, including RAF kinases andPI3K. *KRAS* mutations are highly prevalent in pancreatic intraepithelial neoplasia (PanIN) lesions and are thought to be an early event in PDAC development [48].

**Figure 3:**
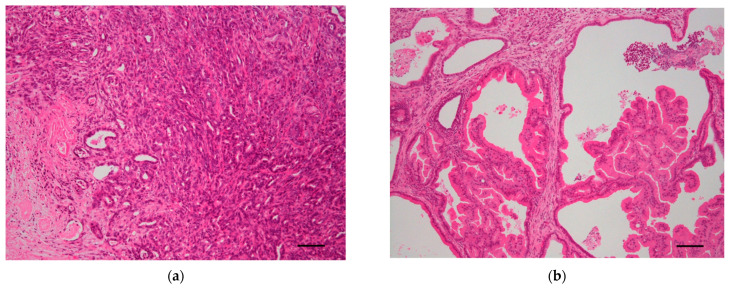

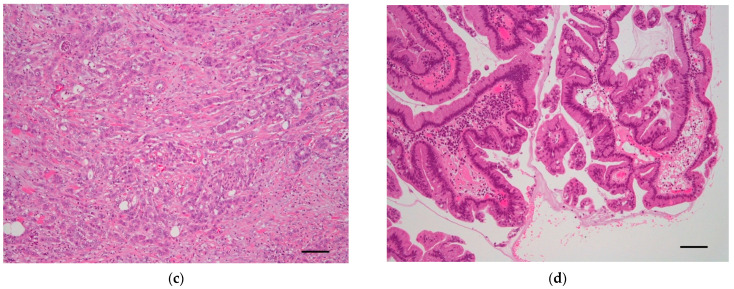
Gross images of genetically engineered mouse models (upper panels) and human pancreatic neoplasms (lower panels), prepared by the authors. (**a**) Developed pancreatic tumor from a *Pdx1*^+/−Cre^; *LSL-Kras*^G12D^ mouse. The tumor consists of an invasive pancreatic ductal adenocarcinoma with a glandular structure. (**b**) Pancreatic tumor from a Tg(*CAG-LSL-GNAS*^R201H^);*LSL-Kras*^G12D^;*Ptf1^Cre/−^* mouse. The tumor consists of dilated ducts with prominent proliferation of epithelial cells that showed complex papillary projections. (**c**) Human invasive ductal adenocarcinoma. (**d**) Human intraductal papillary mucinous neoplasm. Scale bars: 100 μm.

Grippo et al. generated transgenic mice carrying an *Ela^5^-Kras^G12D^* transgene, which targets acinar cells of the pancreas. Two lineages of the founder mice displayed preinvasive pancreatic neoplastic lesions with ductal morphology, suggesting that *Kras* mutations are associated with early stage ductal cancer development [19]. Hingorani et al. crossed mice with the *LSL-Kras^G12D^* allele and Cre knock-in mice at the *Ptf1a* locus (*Ptf1a-Cre*), or mice expressing Cre recombinase under the Pdx1 promoter (*Pdx1-Cre*). Pdx1 and Ptf1a are critical transcription factors involved in pancreatic development. Mutant mice (*PDX1-Cre*;*LSL-Kras^G12D^*, or *P48^+/−Cre^*; *LSL-Kras^G12D^*) developed ductal lesions similar to human PanINs. At low frequency, these lesions progressed to invasive and metastatic PDAC within one year [20]. The neoplasm emerged in this mouse model strongly resembled invasive PDAC in humans. To illustrate phenotypic similarity, representative histological findings observed in the specimen from our library derived from genetically engineered mouse models and human pancreatic neoplasms are shown in Figure 3a,c.

These results suggest that KRAS activation might be sufficient to initiate PDAC carcinogenesis. However, there is a concern that these models reflect developmental defects rather than carcinogenesis, because cancers occur in adult tissue. To induce Kras activation in adult ductal cells or acinar cells, Lee et al. crossed mice with the *Kras*^LSL-G12D^ allele and mice with the tamoxifen-inducible *Sox9CreER* transgene or the *Ptf1a^CreER^* allele. They found that Kras^G12D^ expression in ductal cells induces few PanIN lesions, while Kras^G12D^ expression in acinar cells induces numerous PanIN lesions. These findings indicate that the cellular context where *Kras* mutations are acquired has a significant impact on PanIN initiation. Guerra et al. generated *Kras^G12Vgeo;Elas^-tTA/tetO-Cre* mice, which allowed the controlled temporal expression of an endogenous K-Ras^G12V^ in acinar and centroacinar cells. In this model, adult mice refractory to K-Ras^G12V^-induced PanINs and PDAC differed from the mouse models demonstrated by Lee et al. [18]. However, if challenged with chronic pancreatitis induced by caerulein, they developed PanINs and PDAC [21]. This result suggests that the effects of oncogenic Kras are different between embryonic and adult pancreata, and inflammation is necessary for Kras-induced carcinogenesis in the adult pancreas.

Singh et al. also suggested the importance of inflammation in PDAC development [22]. They generated mice with CAG (CMV and chicken-β-actin chimeric promoter) driven *LGL (lox-GFP-lox)-KrasG12V* allele (cLGL*-Kras^G12V^*) and crossed them with mice harboring *Hnf1b/CreER^T2^* to control the recombination rate depending on tamoxifen dosage in adult pancreatic ducts. *Hnf1b/CreER^T2^*; *cLGL-Kras^G12V^* mice treated with a high dose of tamoxifen developed poorly differentiated, aggressive tumors. The authors also found that there was increased fibrous stroma and inflammation as a higher number of cells expressed oncogenic *Kras* in pancreatic ducts, and suggested that significant duct obstruction may be present in advanced PanIN, which promotes widespread pancreatitis [22].

A single Cre-mediated oncogenic Kras activation does not allow the genetic modeling and manipulation of sequential multistep tumorigenesis. Several dual recombinase mouse models using Pdx-Flp recombinase have been developed to validate possible target genes. Schönhuber et al. generated an inducible dual-recombinase system by combining flippase-*FRT*(Flp*-FRT*) and Cre-*loxP* recombination technologies to reveal the importance of Pdkp1, a downstream effector of PI3K, in PDAC progression. In *Pdx1-Flp*; *FSF-Kras^G12D/+^*; *FSF-R26^CAG-CreERT2^*; *Pdpk1^lox/lox^* mice, Cre-induced Pdkp1 inactivation retained the normal pancreatic tissue architecture with only sporadic PanIN, suggesting that Kras^G12D^-driven PDAC progression depends on intact Pdpk1 expression [23]. Wu et al. generated a *Pdx1-FlpO* knock-in allele, which expresses Flp recombinase in pancreatic epithelial cells. When combined with the *Frt-STOP-Frtkras^G12D^* and *p53^Frt^* mouse lines, activation of mutant Kras and depletion of p53 resulted in the development of PanIN and PDAC [24]. These models provide excellent tools to investigate multistep carcinogenesis and study the roles of genes in different cell types within the tumor microenvironment.

B-RAF is a member of the RAF kinase family that regulates the MAPK signaling pathway. Mutation-activated *BRAF* is detected in melanoma (70%), colorectal cancer (15%), papillary thyroid cancer (40%), ovarian cancer (30%), and non-small-cell lung cancer (3%) [49,50]. *BRAF*^V600E^ mutations are occurring at a low frequency (3%) of PDAC cases. *BRAF* and *Kras* mutations are mutually exclusive [47]. *PIK3CA* encodes the p110α catalytic subunit of PI3K. *PIK3CA* mutations are common in multiple human cancers, including colon (32%), brain (27%), stomach (25%), breast (8%), and lung (4%) cancer [51]. The H1047R mutation in *PIK3CA* is the most common among all solid tumors [52]. This mutation constitutively activates the PI3K pathway, resulting in the phosphorylation of multiple downstream targets. The frequency of *PIK3CA* mutations in PDAC is less than 1%. However, somatic *PIK3CA* mutations have been commonly reported in the ITPNs of the pancreas, which is a rare subtype of premalignant pancreatic lesion, distinct from intraductal papillary mucinous neoplasm (IPMN) [17,53].

*Pdx1-CreERT2*; *Braf^CA/+^* mice were developed by Collison et al., in which expression of BRAF^V600E^ was induced in the adult pancreas under the control of a conditionally active Cre recombinase driven by the Pdx1 promoter [25]. Pancreatic expression of BRAF^V600E^ led to near-total replacement of the exocrine pancreas with PanIN lesions. The authors also developed *Pdx1-CreER^T2^*; *Pik3ca^lat-H1047R^* mice in which mutationally activated *PIK3CA^H1047R^* was expressed from the endogenous *Pik3ca* locus after Cre-mediated recombination. However, detectable PanIN lesions or other pancreatic abnormalities were not found in these mice up to six months after Cre induction. These data indicate that mutationally activated BRAF^V600E^, but not PIK3CA^H1047R^, can initiate PanINs in this mouse model [25]. Payne et al. generated two murine models that expressed a constitutively active PIK3CA within the pancreas [26]. They crossed *Pdx1-Cre* transgenic mice with Pik3ca^p110*^ mice which carried a conditional allele encoding an active PIK fusion protein, or *Pik3ca^H1027R^* mice. In the first model, PanINs were detected as early as 10 days of age and invasive pancreatic ductal adenocarcinoma developed as early as 20 days of age. In the second model, PanINs and invasive cancer developed with greater latency due to a lesser degree of PI3K pathway activation. These *Pik3ca* mutant pancreatic cancers were shown to be morphologically indistinguishable from *Kras* mutant models, demonstrating the importance of PI3K signaling in the oncogenic potential of pancreatic cancer. These cancers also showed activation of ERK1/2 signaling, which might be downstream of PI3K signaling, as it occurs early in tumorigenesis [26].

PTEN (phosphatase and tensin homolog) is a member of the tyrosine phosphatase type I family and an important tumor suppressor. In the active phase, PTEN is dephosphorylated at its C-terminal portion, and is recruited from the cytosol to the membrane, thereby preventing the hydrolysis of PIP2 to PIP3. Thus, PTEN inhibits the PI3K signaling pathway [54]. *PTEN* deficiency causes hyperactivity of the PI3K pathway owing to the accumulation of PIP3. *PTEN* loses its function through genetic mutations, posttranslational modifications, and epigenetic mechanisms. *PTEN* germline mutations are seen in some syndromes, such as PTEN hereditary tumor syndromes, Cowden syndrome, Bannayan—Riley—Ruvalcaba syndrome, and Proteus syndrome. Furthermore, they develop benign tumors in various organs and cancers of the thyroid, prostate, or breast [55].

Kopp et al. generated mice with Pten disruption specifically in adult pancreatic ducts [27]. These mice developed IPMNs (pancreatobiliary, and oncocytic), and 31.5% of IPMNs became invasive. The invasion was associated with the pancreatobiliary subtype and spontaneous mutations in *Kras* [27].

These results indicate that the RAF/MEK/ERK and PI3K/AKT signaling pathways play a central role in the initiation and development of PDAC.

### 2.2. MYC Activation

MYC belongs to the family of the basic helix-loop-helix-leucine zipper transcription factor and regulates cell growth, differentiation, and metabolism. Its expression is tightly controlled in normal cells but it is overexpressed in human cancers [56]. Pancreatic acinar cell carcinomas are distinct aggressive neoplasms in which *MYC* amplification is frequent (17%) [57]. MYC expression was also increased in a subset of PDAC [58]. Several studies have suggested the important roles of MYC in PDAC cell maintenance. Ying et al. suggested that Myc is an essential mediator of Kras-induced metabolic changes in pancreatic cancer cells [59]. Lin et al. revealed that Myc cooperates with PIN1 and induced NRF2 expression to counteract KRAS-induced mitochondrial respiratory injury in pancreatic cancer cells [60].

Sandgren et al. showed that expression of Myc under the elastase (Ela) promoter can induce mixed acinar/ductal pancreatic adenocarcinomas between 2 and 7 months of age [28]. Lin et al. developed a new model that allows the temporally and spatially controlled expression of Myc in pancreatic progenitors and derived lineages of exocrine cells The authors demonstrated that upregulation of Myc alone led to the initiation of ductal precursor lesions and the formation of ductal adenocarcinomas after a short latency [29]. They also showed that, following the ablation of Myc, despite a macroscopically complete regression of primary and metastatic tumors, some cancer cells remained dormant, and that re-expression of exogenous Myc in these cells led to rapid cancer recurrence. This study highlights the importance of novel eradication strategies for residual cancer cells. Rajbhandari et al. used mice expressing Myc or Kras^G12D^ in a doxycycline-controlled manner in the pancreas to perform a genome-wide gene expression analysis after ablation of these oncogenes to reveal a survival mechanism. They identified an increase in autocrine IGF1/AKT signaling in dormant cancer cells, and showed that pharmacological inhibition of IGF-1R reduces residual tumor and cancer recurrence [30].

Maddipati et al. developed the KPCXY (*Pdx1CreER*; *Kras^G12D^*; *Trp53^fl/+^*; *Rosa^confetti/YFP^*) model, employing multiplexed fluorescence-based labeling to track the multiple primary tumor cells lineages during metastasis [31]. They found that enhanced expression of Myc promotes metastatic spread by recruiting tumor-associated macrophages and single cell analysis of a paired primary and metastatic tumor of PDAC patients, and revealed the enrichment of MYC-amplified subclones in metastatic lesions compared to the primary cells [31].

### 2.3. IPMN Related Pathways

**Figure 4:**
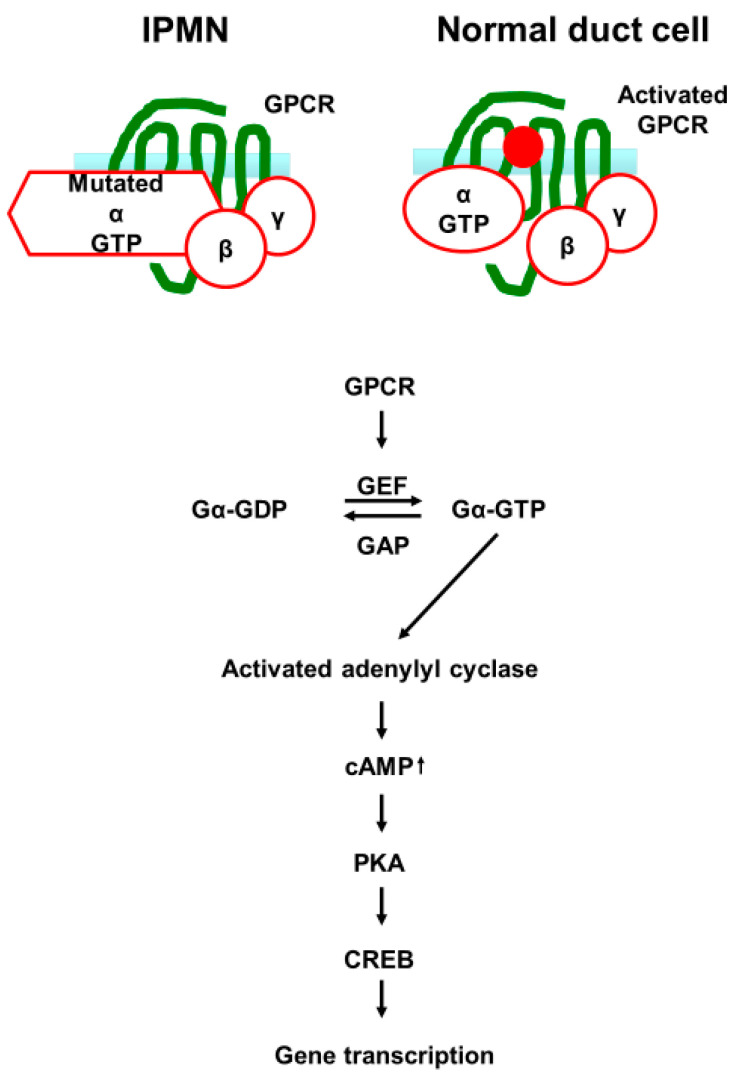
Schematic sequences of G protein activation after G protein-coupled receptors (GPCR) binding to its ligand. G-proteins are composed of three subunits: α, β and γ. Ligand-activated GPCR allows the release of GDP from G proteins and causes the exchange of GDP to guanosine triphosphate (GTP) in the α subunit. The GTP-bound α subunit dissociates from the β–γ complex and activates effector proteins. This results in the activation of adenylyl cyclase, which produces the cyclic adenosine monophosphate (cAMP) that activates protein kinase A (PKA). PKA directly activates the cAMP response element-binding (CREB) which induces target gene transcription. These activations continue until the GTP is hydrolyzed by the intrinsic GTP hydrolysis activity. GNAS mutations observed in IPMNs cause disruption of the intrinsic hydrolytic activity of Gsα, which results in constitutive activation of adenylyl cyclase.

The G protein-coupled receptor (GPCR) family comprises more than 800 members. After binding to their ligands, heterotrimeric G proteins are activated and generate secondary messengers. Kinase cascades are activated in the cytoplasm of the cells. These signals ultimately control gene transcription, cell survival, motility, and growth. When bound to GTP, G proteins are active. However, an intrinsic GTPase activity allows their inactivation in the CDP-bound status (Figure 4). The *GNAS* gene family contains several G protein members, including *GNAS*, *GNA11*, and *GNAQ*, which encode the Gαs, Gα11, and Gαq subunits, respectively [61]. Oncogenic mutations in these genes impair their GTPase activity, leading to constitutive GTP-bound active forms and extended downstream signaling (Figure 4) [62]. Mutations in GNAS occur in a wide range of tumors. The most frequently reported entities (10%) are colorectal and stomach tumors [63].

IPMNs are among the precursor lesions of PDAC. Genomic specificity and mutations of the *GNAS* gene have been described [64]. PDAC derived from an IPMN is termed an IPMN-associated carcinoma and is defined as a subgroup. Microscopically, IPMNs are characterized by dilated mucinous pancreatic ducts lined by columnar mucin-producing cells that show papillae with fibrovascular cores. The common *GNAS* mutations observed in IPMNs are R201C and R201H. Using a unique ligation assay, Wu et al. analyzed 132 IPMNs (113 IPMN tissue samples and 19 cyst fluids) and found that 66% of IPMNs harbored *GNAS* mutations, 81% harbored *KRAS* mutations, and 51% harbored both *GNAS* and *KRAS* mutations [65]. Kuboki et al. found that 48% of 172 IPMNs harbored *GNAS* mutations, 56% harbored *KRAS* mutations, and 31% harbored both *GNAS* and *KRAS* mutations [66]. *GNAS* mutations were observed in both low-grade and high-grade tumors as well as in invasive tumors. In contrast, *GNAS* mutations are not found in conventional PDACs [64,65]. The *GNAS* mutation probably plays a crucial role in the initiation, rather than progression, of the pathogenesis of IPMN. IPMNs are divided into gastric, intestinal, and pancreatobiliary subtypes [67]. *GNAS* mutations are significantly associated with the intestinal subtype (60% of cases) [66].

Interestingly, *GNAS* and *KRAS* mutations have also been detected in another mucinous neoplasm termed pseudomyxoma peritonei (PMP) [68]. PMP is a rare clinical malignancy characterized by uncontrollable accumulation of mucinous ascites in the peritoneal cavity. GNAS mutations are the main contributor to mucin hypersecretion. Nishizawa et al. demonstrated that *GNAS* mutations might regulate mucin production through the cAMP-protein kinase A (PKA) pathway [69]. The activated cAMP-PKA signaling pathway might stimulate cAMP-response element-binding protein (CREB) and activating transcription factor (ATF) family. CREB/ATF then combines with the upstream cis-acting element of mucin genes and promotes mucin expression.

Taki et al. generated mice genetically engineered to conditionally express mutated GNAS in the pancreas [32]. The mice displayed microscopic dilation of the pancreatic ducts and parenchymal fibrosis by 2 months of age. However, when crossing *Tg(CAG-LSL-GNAS*^R201H^*)*;*Ptf1a^Cre/+^* mice with LSL-*Kras*^G12D^ mice, generating *Tg(CAG-LSL-GNAS*^R201H^*)*;*LSL-Kras^G12D^*; *Ptf1a^Cre/+^* mice, within 5 weeks the *Tg(CAG-LSL-GNAS*^R201H^*)*;*LSL-Kras*^G12D^;*Ptf1a^Cre/+^* mice developed a cystic tumor consisting of marked dilated ducts lined with papillary dysplastic epithelia in the pancreas, which closely mimics human IPMN (Figure 3b,d). These data strongly suggest that activating mutations in *GNAS* and *KRAS* cooperatively promotes murine pancreatic tumorigenesis, which resembles human IPMN [32].

*LKB1/STK11* (liver kinase B1/serine-threonine kinase 11) encodes a ubiquitously expressed serine-threonine kinase and positively regulates downstream kinases that are involved in the regulation of cellular response to energy stress and the establishment of cell polarity [70]. *LKB1* was first identified as a tumor suppressor gene associated with Peutz–Jegher Syndrome (PJS), a rare autosomal dominant syndrome. Most cases of PJS (>80%) are caused by germline mutations in the *LKB1* gene. Individuals with PJS are at an increased risk of developing cancers in multiple organs [71] and harbor a 132-fold increased risk of pancreatic cancer [72]. Deleterious mutations of *LKB1* are found in 4–25% in IPMN [73,74,75]. Some studies suggested that pancreatic cancers in patients with PJS might arise from the IPMN [76,77]. These facts suggest that synergism between *Kras^G12D^* and *LKB1* mutations may lead to IPMN in the pancreatic ducts. Collet et al. created a mouse model in which mutations in *Kras* and *Lkb1* were conditionally induced in adult pancreatic ducts. The authors demonstrated that activating *Kras^G12D^* mutation and *Lkb1* inactivation induced IPMN, mainly of the gastric type, and shared several features with human IPMN [33].

These results suggest that oncogenic GNAS activation and LKB1 inactivation are involved in the initiation of IPMN cooperating with mutant KRAS.

### 2.4. SMAD4

Mothers against decapentaplegic homolog 4 (SMAD4) belongs to the SMAD family of transcription factor proteins which mediate TGF-β, morphogenetic protein (BMP), and activin signal transduction. Ligands bind to the serine/threonine kinase receptor and to the phosphorate receptors SMADs-SMAD1, SMAD2, SMAD3, SMAD5, and SMAD8. Receptor SMADs bind to SMAD4, translocate to the nucleus and regulate the expression of broad sets of genes [78]. TGF-β is a potent inhibitor of epithelial cell growth, although its effects are highly dependent on cellular content [79]. The tumor suppressive role of TGF-β signaling is suggested by the presence of inactivated TGF-β receptor mutations in several cancers. In contrast, TGF-β also promotes tumor cell proliferation, migration, and epithelial-to-mesenchymal transition (EMT) in some established epithelial tumors [80]. Therefore TGF-β signaling may have biphasic roles, inhibiting carcinoma initiation while promoting the high-grade advancement of established tumors. Inactivating mutations in *SMAD4* are far more common in PDAC (31%) than in other cancer types [81,82,83], and are generally associated with high-grade PanIN lesions [84].

In MCN, genetic inactivation of *SMAD4* may occur late in the neoplastic progression of MCN. *SMAD4* mutations are not observed in most noninvasive MCNs, but protein expression is frequently lost in invasive cancers arising from MCNs [85].

Bardeesy et al. generated mice with a conditional knockout allele of *Smad4* (*Smad4^lox^*) and crossed them with mice harboring either *Pdx1-Cre* or *Ptf1a-Cre*. The combination of *Kras^G12D^* and *Smad4* deficiency accelerated the progression of PanINs and the rapid development of tumors resembling human gastric type IPMN. *Smad4* deficiency also accelerates PDAC development in KIC mice [34].

### 2.5. CDKN2A (p16INK4 and p14ARF)

Cyclin-dependent kinase inhibitor 2A gene (*CDKN2A*) encodes two different proteins (p16INK4 and p14ARF) by sharing exons 2 and 3 with distinct reading frames. Both p16INK4 and p14ARF regulate the cell cycle and act as tumor suppressor genes. P16INK4 inhibits the cyclin-dependent kinases, including CDK4 and CDK6, and triggers the phosphorylation of RB protein resulting in cell cycle arrest at the G1-to-S phase. The other protein of *CDKN2A*, p14AFR, inhibits MDM-2, a negative regulator of p53, and controls cell cycle progression [86,87].

*CDKN2A* is a widely studied and commonly mutated gene in various cancers. The *CDKN2A* gene is inactivated in most PDAC cases (98%), caused by several different mechanisms, such as homozygous deletion, inactivating mutation in one allele with loss of heterogeneity, or promoter hypermethylation [88]. Inactivation of *CDKN2A* occurs in 40% of PanIN lesions [89,90].

Mice with constitutive deletion of the *P16Ink4a/P19Arf* locus did not develop spontaneous pancreatic cancer [91]. Aguirre et al. generated mice with pancreas-specific Kras^G12D^ expression and homozygous deletion of *P16Ink4a/P19Arf* (KIC) mice. These mice developed highly aggressive PDAC with frequent EMT [35].

### 2.6. TP53(p53)

*TP53 (Tumor protein 53)*) is the most widely characterized tumor suppressor gene in cancer. p53 is activated via posttranslational alterations in response to multiple cellular stresses, such as oncogenic activation, DNA damage, and hypoxia. These factors result in the activation of p53, which triggers multiple cascades, such as G2-M arrest, DNA repair, metabolic changes, cellular senescence, and apoptosis. Activated p53 transactivates downstream target genes involved in these cellular processes [81]. *TP53* is recognized as a commonly altered gene in pancreatic cancer. Whole-genome sequencing analysis revealed that the prevalence of inactivation events for *TP53* was 74% (three structural variants and 71 mutations in 100 cases) [82].

Several mouse models have been developed by a combination of oncogenic Kras and inactivation of p53. Hingorani et al. generated a conditionally expressed *Trp53^R172H^* mutant, which is related to Li–Fraumeni syndrome. After crossing with Pdx1-Cre mice, activation of both *Kras^G12D^* and *Trp53^R172H^* alleles occurred in pancreatic progenitor cells. Mice with both mutant *Kras^G12D^* and *Trp53^R172H^* (KPC mouse) developed spontaneous PDAC and died of cancer within 1 year [36].

*TP53* mutations arise as late events in advanced human IPMN [74]. To examine the impact of *p53* alteration, *Patra* et al. generated mice with conditional heterozygous Kras^G12D^, Gnas^R201C^, and Tp53^Loxp/+^ alleles and crossed them with mice harboring Ptf1a-Cre^ER^ to establish KGPC^ER^ (*Kras^G12D^*;*Gnas^R201C^*;*p53^loxP/+^*;*Cre^ER^*) mice. KGPC^ER^ mice following Cre recombinase induction developed invasive tumors and malignant ascites with short latency (mean 25.8 weeks), and a histological analysis of the mice revealed PDACs contiguous with high-grade IPMNs and showed liver and peritoneal dissemination. Loss of wild-type *Tp53* was observed in three of the four PDAC tumors of KGPC^ER^ mice. These data suggested *Tp53* inactivation enables IPMN-to-PDAC progression [37].

### 2.7. Homologous Recombination Deficiency

BRCA1 (breast cancer type 1 susceptibility protein) and BRCA2 are tumor suppressor proteins involved in repairing double-strand breaks via homologous recombination. Homologous recombination is a complex mechanism involving many other proteins, including PALB2 (partner and localizer of BRCA2), ATM (ataxia telangiectasia mutated) and RAD50. Alterations in the BRCA pathway result in homologous repair deficiency (HRD). Deleterious mutations of BRCA were identified as a risk factor for the development of breast and ovarian cancer. BRCA is a risk factor for PDAC. Deleterious germline mutations in *BRCA1* and *BRCA2* are found in patients with both familial PDAC (FPC) and non-familial PDAC [92]. In FPC patients, the frequency of *BRCA2* may be as high as 17% [93,94,95,96], and *ATM* and *PALB2* mutations are approximately 2.4% and 1 to 4.9%, respectively [97].

Cancers harboring HRD may be susceptible to drugs that induce double-strand breaks in DNA. The efficacy of platinum therapy has been established for breast and ovarian cancers with HRD. There is growing evidence that PDAC with HRD is also beneficial. Recently, poly (ADP-ribose) polymerase (PARP) inhibitors have emerged as a novel class of targeted therapies for HRD-tumors. PARP is an important protein for the repair of single-strand breaks. PARP inhibitors bind to the catalytic domain of PARP and trap it to the single-strand DNA break, leading to the accumulation of double-strand breaks. Therefore, HRD-tumor cells undergo cell cycle arrest and apoptosis when exposed to PARP inhibitors. The National Comprehensive Cancer Network guidelines recommend universal germline testing for all patients with PDAC, given the significant treatment implications they may have [97].

Drosos et al. developed *LSL-Kras^G12D^*; *Ptf1a^+/cre^*; *ATM^loxP/loxP^* mice and demonstrated that ATM deficiency synergizes with Kras^G12D^ to promote the formation of highly metastatic pancreatic tumors [38]. PDAC mouse models based on BRCA1 or 2 mutations have not been reported to date.

### 2.8. WNT Signaling

*RNF43*(ring finger protein 43) encodes a transmembrane E3 ubiquitin ligase [98] that downregulates the Wnt/β-catenin pathway by ubiquitinating the Wnt receptor and exerting tumor suppressor activity [99]. Mutations in RNF43 have been reported in several neoplasms, including colorectal cancer (18.9%) and endometrial cancer (18.1%). Most of the mutations are truncating mutations [100]. Sakamoto et al. uncovered somatic *RNF43* mutations in eight (14%) of 57 IPMNs, in which all the mutations were supposed to be loss-of-function mutations [101]. Wu et al. found that six of eight IPMNs and three of the eight MCNs harbored mutations of RNF43 [102], suggesting that RNF43 is a tumor suppressor for both IPMNs and MCNs. Mishra et al. showed that disruption of *RNF43* accelerates *Kras*^G12D^ dependent tumorigenesis using inducible CRISPR/Cas9 gene editing. However, IPMNs did not develop [39].

### 2.9. Chromatin Regulation Related Genes; ARID1A, BRG1 and KMT2C

Chromatin regulation may play an important role in PDAC in addition to the core pathways described above. Two chromatin remodeling complexes are affected by inactivating mutations in the SWI/SNF complex and the COMPASS (complex of proteins associating with Set1) complex [103,104]. Inactivation of genes in the SWI/SNF or COMPASS complexes has been correlated with basal-cell subtypes and worse outcomes [7].

*ARID1A* encodes a protein of the PBAF subunit of the SWI/SNF complex [105]. Somatic mutations of *ARID1A* in pancreatic cancer were initially identified by whole-genome sequencing [6], and validated in a larger series of human tumors. Most mutations were truncating, with the frequency of mutations being highest in the colon (10%), stomach (10%), and pancreas (8%) [106]. Interestingly, the mutations of individual SWI/SNF subunits occurred at the modest frequency in PDAC, but together they affected at least one-third of all pancreatic cancers, suggesting that SWI/SNF is a central tumor suppressive complex in PDAC [107]. Kimura et al. generated *ptf1a-Cre*;*Kras^G12D^* mice with conditional disruption of Arid1A. These mice developed IMPN-like tumors and PDACs. [40]. Wang et al. evaluated tumors developed in *Ptf1a-Cre*;*Kras^G12D^*;*Arid1a^f/f^* mice and showed that the EMT and stem cell identity pathways were activated in these tumors [41].

*BRG1*, which is inactivated in PDAC, is a core subunit of SWI/SNF, also known as SMARCA4 [6,107]. BRG1 expression was reportedly frequently reduced or lost in human IPMN samples [108]. von Figura et al. crossed mice carrying floxed alleles of *Brg1* with *ptf1-Cre*; *Kras^G12D^* mice. They found that the loss of Brg1 cooperates with oncogenic Kras to form cystic neoplastic lesions that resemble human IPMN and progress to PDAC. Although Brg1-null IPMN-PDAC develops rapidly, it is less lethal than PanIN-PDAC driven by mutant *Kras* and hemizygous *p53* deletion [42].

Of note, mice with inactivated Arid1A or Brg1 in the pancreas developed a similar type of cystic tumor resembling human IPMN in cooperation with oncogenic Kras. These results suggest that deficiency of the SWI/SNF complex is involved in the IPMN-PDAC carcinogenesis pathway. More studies are needed to explore the downstream targets of the SWI/SMF complex involved in IPMN precursor development.

*KMT2C* belonging to the TRX/MLL gene family encodes a histone lysine methyltransferase, a subunit of the COMPASS complex. Jones et al. identified *KMT2C* as a common mutational target in PDAC [11]. Mann et al. established an oncogenic LSL-KRAS^G12D^ mouse coupled with the sleeping beauty transposon system to screen for mutations, in combination with Kras-derived tumorigenesis. Using this screening system, *KMT2* was identified as one of several candidate genes involved in PDAC development [109].

These data indicate an important and emerging role for chromatin remodeling in PDAC development. However more intensive studies are required to determine how it works at the molecular level.

## 3. Discussion and Future Directions

**Figure 5:**
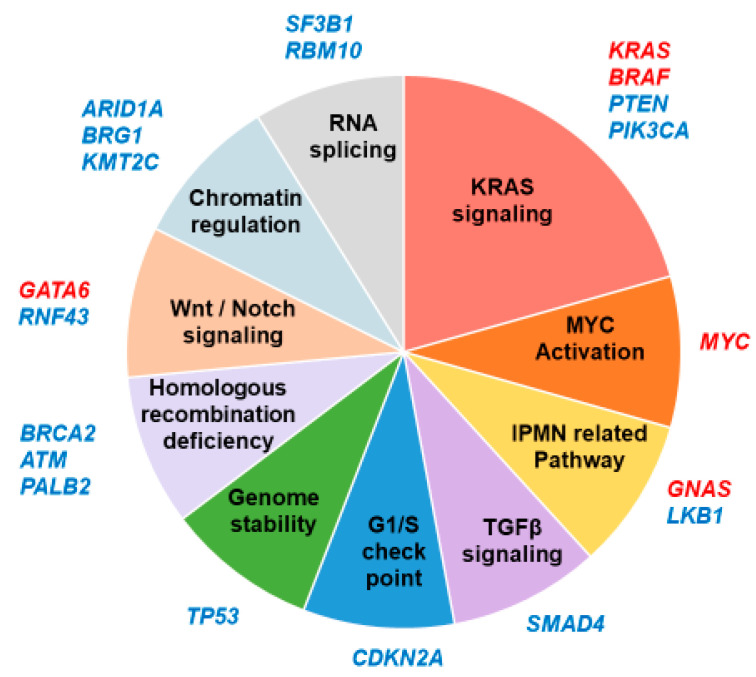
Commonly altered driver genes in PDAC organized by molecular function. The activated genes are written in red, and the inactivated genes are written in blue.

In this review, we have focused on the commonly altered driver genes of PDAC and their related GEMMs, recapitulating the histological hallmarks of human pancreatic neoplasms (Figure 3). GEMMs have revealed the crucial roles of drivers during development of PDAC [110]. Our systematic review of mouse GEMMs prompted us to categorize the commonly altered genes into 10 groups; KRAS signaling, MYC activation, IPMN related pathway, TGFβ signaling, G1/S checkpoint, homologous recombination deficiency, DNA damage repair, Wnt/Notch signaling, chromatin regulation, and RNA splicing (Figure 5). One crucial difference in this classification compared to that in the previous categorization proposed by Hayashi et al. was our characterization of IPMN-related genes as an independent gene family. We believe that mutation in GNAS is rather specific to IPMN, as we have previously demonstrated [64], and it deviates from the KRAS pathway. Mutation in LKB1 is highly likely to be associated with IPMN, as Collet et al. indicated in the literature [33].

Among the frequently altered genes listed in the Figure 5, *GATA6*, *SF3B1*, and *RBM10*, were not discussed in this review because mouse models of PDAC based on their mutations have not been established to date.

GATA6 is a transcription factor involved in the normal development of various organs, including the pancreas [111]. In PDAC, *GATA6* amplification or transcriptional upregulation occurs late during carcinogenesis to activate canonical Wnt signaling [112]. *GATA6* amplification or overexpression correlates with the classical molecular subtype, *SMAD4* deletion, and improved overall survival [113]. Loss of GATA6 expression is correlated with the basal-like molecular subtype and poor prognosis [8]. Martinelli et al. reported that pancreas-specific Gata6 ablation rendered acinar cells more sensitive to Kras^G12V^-driven pancreatic tumorigenesis and suggested that Gata6 exerted its tumor suppressive effect by promoting cell differentiation and suppressing inflammatory pathways [114]. Gata6 overexpressing PDAC mouse models are needed to understand their oncogenic function during PDAC development.

SF3B1 and RBM10 have roles in mRNA splicing and mutations in their genes recurrently identified in PDAC [115]. SF3B1 is a subunit of the U2 snRNP, which recognizes the branch point adenosine base within the intron. SF3B1 is the most commonly mutated splicing factor in cancer. *SF3B1* mutational hot spots may affect protein–protein interactions, likely resulting in changes in function. For RBM10, a member of the RNA-binding motif gene family, inactivating nonsense or frameshift mutations is common, resulting in loss of function [116]. Obeng et al. generated mice with the conditional allele of *Sf3b1^K700E^* knocked-in. After activation of the K600E mutation, these mice showed progressive macrocytic anemia, and the progenitors of these mice demonstrated aberrant 3′ splice-site selection associated with increased nonsense-mediated decay [117]. Experiments of crossing these mice with KC mice would be useful to reveal the roles of splicing dysfunction during pancreatic cancer development, which may be a novel therapeutic target.

Next-generation sequencing revealed an emerging number of genetic alterations. GEMMs have proven to be a powerful tool for studying the impact of gene function on tumorigenesis. However, it is expensive and time-consuming to generate conventional GEMMs based on each mutation. To address this problem, Saborowski et al. developed flexible embryonic stem cell-based GEMMs. These stem cells harbor a latent Kras mutant, a homing cassette, and gene elements needed for rapid insertion and conditional expression of tetracycline-controlled transgenes [118]. In combination with short hairpin RNA or gene editing technology, this system provides the means to investigate the role of multiple candidate genes simultaneously on a time scale of months.

Recently, GEMMs expressing fluorescent proteins to trace tumor cells have been developed. Therapeutic tests using GEMM pancreatic cancer models have been an important step towards drug development in preclinical studies, although there is variability in tumor initiation, progression, and incidence of metastasis in KPC mice. Advanced cell tracing approaches have been employed to monitor tumor growth and metastasis. Oca et.al. crossed KIC mice with RGS16::green fluorescent protein (GFP) transgenic mice. RGS16::GFP is a Kras-dependent tumor reporter that correlates with tumor burden. The effects of gemcitabine and receptor tyrosine kinase Axl inhibitors were observed by the reduction of GFP expression. This in vivo model provides a highly sensitive screening tool for tumor-inhibiting molecules [119].

Lineage tracing approaches are useful tools for detecting the unique attributes of each heterogeneous tumor cell. Rhim et al. developed a tag-and-track KPC model in which yellow fluorescence protein (YEP) labeled tumor epithelial cells undergoing EMT could be monitored. These YEP-labeled cells were observed in circulation at an early stage, even before the development of invasive cancer in the pancreas [120]. Maddipati et al. developed KPCX mice in which a tamoxifen-inducible Cre recombinase (C) simultaneously activates an oncogenic Kras^G12D^ allele (K), deletes a single p53 allele (P), and generates a color-producing recombination event within the Rosa^Confetti^ locus (X). Using KPCX mice, they demonstrated that significant fractions of metastases were polyclonally seeded by distinct tumor subclones, leading to either monoclonal or polyclonal expansion differing on the site of metastasis [121].

These newly developed approaches will accelerate the understanding of the roles of genetic events in PDAC progression for the future development of novel therapies.

## 4. Conclusions

We have highlighted the clinically significant mutated genes that are frequently observed in human pancreatic cancers, and comprehensively summarized genetically engineered corresponding mouse models. Based on the systematic analyses of the currently available mouse models, we have attempted to classify those frequently mutated genes into ten categories based on the unveiled function of each gene explored in an individual manner. One can anticipate that these categorized genes their functional analyses will further be updated with the facts derived from the development of new animal models. Precision medicine will be materialized via the screening process of a potentially valid treatment modality by employing those mouse models to the maximum extent.

## Figures and Tables

**Table 1:** Mouse models of pancreatic ductal neoplasms.

Genotype	Time of Expression	Phenotype	Reference
*Sox9CreER*;*Kas^LSL-G12D^*;*Trp53^flox/flox^* *Ptf1aCreER*;*Kras^LSL-G12D^*;*Trp53^flox/flox^*	Inducible	PanIN, PDAC	Lee, 2019 [18]
*Tg(Ela^5^-Kras^G12D^)*	~P30	Preinvasive ductal neoplasia, acinar cell dysplasia	Grippo, 2003 [19]
*Pdx1-Cre*;*LSL-Kras^G12D^*	E8.5	PanIN, PDAC	Hingorani, 2003 [20]
*Ptf1^Cre/+^*;*LSL-Kras^G12D^*	E9.5	PanIN, PDAC	Hingorani, 2003 [20]
*Kras^G12Vgeo^*;*Elas-tTA/tetO-Cre*	Inducible	PanIn, PDAC	Guerra, 2007 [21]
*Tg(CAG-lox-GFP-stop-lox-Kras^G12V^)*;*Hinf1b/CreER^T2^*	Inducible	PanIN, PDAC	Singh, 2021 [22]
*Pdx1-Flp*;*FSF-Kras^G12D/+^*;*FSF-R26^CAG-CreERT2^*	E9.5	PanIN, PDAC	Schönhuber, 2014 [23]
*Pdx1-FlpO*;*Frt-STOP-Frt kras^G12D^*	E9.5	PanIN, PDAC	Wu, 2017 [24]
*Pdx1-CreER^T2^*;*Braf^CA/+^*	Inducible	PanIN	Collisson, 2012 [25]
Tg(Pdx1-Cre)Pik3^cap110*^	E8.5	PanIN, PDAC	Payne, 2015 [26]
*Tg(Pdx1-Cre)*;*Pik3ca^H1047R^*	E8.5	PanIN, PDAC	Payne, 2015 [26]
*Sox9-CreER^T2^*;*Pten^flox/flox^*;*LSL-Kras^G12D^*	Inducible	IPMN, PDAC	Kopp, 2018, [27]
*Tg(Ela-1-myc)*	~P30	Mixed acinar/ductal adenocarcinoma	Sandgren, 1991 [28]
*Pdx1-Cre*;*CAG-tTA*;*TetO-Myc*	Inducible	PanIN, PDAC	Lin, 2013 [29]
*Pdx1-Cre*;*CAG-tTA*;*TetO-Kras^G12D^*	Inducible	PanIN, PDAC	Rajbhandari, 2017 [30]
*Pdx1CreER*;*Kras^G12D^*;*Trp53^fl/+^*; *Rosa^confetti/YFP^*	Inducible	PanIN, PDAC	Maddipati, 2021 [31]
*Tg(CAG-LSL-GNAS^R201H^)*; *LSL-Kras^G12D^*;*Ptf1^Cre/+^*	E9.5	IPMN	Taki, 2016 [32]
*Sox9-CreER^T2^*;*LSL-Kras^G12D^*;*Lkb1^flox/flox^*	Inducible	IPMN	Collet, 2020 [33]
*Pdx1-Cre*; *LSL-Kras^G12D^*; *Smad4^flox/flox^*	E8.5	IPMN, PanIN	Bardeesy, 2006 [34]
*Pdx1-Cre*;*LSL-Kras^G12D^*;*Ink4a/Arf^flox/flox^*;*Smad4^flox/flox^*	E8.5	IPMN, differentiated PDAC	Bardeesy, 2006 [34]
*Pdx1-Cre*; *LSL-Kras^G12D^*; *Ink4a/Arf^flox/flox^*	E8.5	PanIN, pooly differentiated PDAC	Aguirre AJ, 2003 [35]
*Ptf1^Cre/+^*;*LSL-Kras^G12D^*;*LSL-Trp53^R172H/+^*	E9.5	PanIN, PDAC	Hingorani, 2005 [36]
*Ptf1a-CreER*;*LSL-Kras^G12D^*;*Tp53^loxP/+^*;*LSL-Gnas^R201C^*	Inducible	IPMN, PDAC	Patra, 2018 [37]
*LSL-Kas^G12D^*;*ptf1a^+/cr^*^e^;*ATM^loxp/loxP^*	E8.5	PDAC, metastaic	Drosos, 2016 [38]
*Ptf1^Cre/+^*;*LSL-Kras^G12D^*;*rtTA3^lox/lox^*;*sgRnf43*;*Tre3g-Cas9*	Inducible gene editing	PanIN, PDAC	Misha, 2020 [39]
*Ptf1^Cre/+^*;*LSL-Kras^G12D^*;*Arid1a^flox/flox^*	E9.5	IPMN, PDAC	Kimura, 2018 [40] Wang, 2019 [41]
*Ptf1^Cre/+^*,*LSL-Kras^G12D^*;*Brg1^flox/flox^*	E9.5	IPMN, PDAC	Von Figura, 2014 [42]

PanIN: Pancreatic intraepithelial neoplasia; PDAC: Ductal adenocarcinoma of the pancreas; IPMN: Intraductal papillary mucinous neoplasia.
